# Liverpool Child Welfare

**Published:** 1920-06-12

**Authors:** 


### Liverpool Child Welfare.

FORTY-FIVE thousand children have passed through the hands of the Liverpool Child Welfare Association, the scope of whose work is wide. The Association was inaugurated not more than twelve years ago, and now, as the recognised "necessary adjunct" of the Public Health Department of the city, it has linked up a complete scheme for the physical care of child life. Its organisation comprises ante-natal clinics, home helps, an experiment in motherhood subsidies, the physical care of the school child, the following up of school medical inspection, and of the hospital out-patients and in-patients, the provision of convalescent care, of long treatment in country hospitals, of tonics, milk, surgical apparatus, and of any aid necessary for the cure and prevention of disease. After-care supervision and recreation were also included in the scheme. The report declares that the success of the effort depends upon the extent to which it is correlated to that centre of the child's existence—its home. It is in the home visiting and supervision that the success or failure of child welfare stands. The parents are contributing in increasing measure towards the cost of the treatment necessary for their children, and in 1919 their contributions were over £5,600, but there is a debit balance of nearly £5,000. That the value and efficiency of the work are recognised was shown by the fact that promises of £1,250 were made at the annual meeting held last week under the presidency of the Lord Mayor.

